# Congenital adrenal hyperplasia due to 11-Beta-hydroxylase deficiency in a Tunisian family

**DOI:** 10.11604/pamj.2020.36.226.24270

**Published:** 2020-07-28

**Authors:** Hamza Elfekih, Asma Ben Abdelkrim, Hajer Marzouk, Ghada Saad, Moez Gribaa, Yosra Hasni, Amel Maaroufi

**Affiliations:** 1 Department of Endocrinology-Diabetology, Farhat-Hached University Hospital, Sousse, Tunisia,; 2 Department of Cytogenetic and Reproductive Biology, Farhat-Hached University Hospital, Sousse, Tunisia

**Keywords:** Adrenal hyperplasia, congenital, 11-beta-hydroxylase deficiency, hypertension, hypokalemia, metabolic syndrome

## Abstract

Congenital adrenal hyperplasia refers to a group of rare genetic disorders affecting the adrenal glands. 21-hydroxylase deficiency is the most prevalent and the most studied cause while the remaining enzymatic defects are less common, accounting for less than 10% of cases. We herein described the clinical, biological and molecular characteristics and outcome of patients of the same family diagnosed with 11-Beta-hydroxylase deficiency. The disorder was revealed by peripheral precocious puberty between the age of 2-3 years in males and by the virilization of the external genitalia in females. Genetics finding a homozygous p.Gly379Val mutation in the CYP11B1 gene. All patients received hydrocortisone supplementation therapy and mineralocorticoid-receptor antagonist. The females underwent a surgical correction of the ambiguous genitalia at the neonatal age. Long term follow-up revealed metabolic syndrome, obesity and hypertension in the first two patients, an impaired final height in the two females and hypokalemia in three patients.

## Introduction

Congenital adrenal hyperplasia (CAH) is an inherited autosomal recessive genetic endocrine disease. It regroups several disorders resulting from the deficiency of one of the steroidogenesis enzymes. 21-hydroxylase deficiency (21-OHD) is the most common accounting for 90-99% of CAH cases followed by 11-Beta-hydroxylase deficiency (11β-OHD) accounting approximately for 0.2-8% [1]. The incidence of 11β-OHD is estimated at 1: 100 000 live births in nonconsanguineous populations, and can be as high as 1: 5000 in the Moroccan Jewish population [2,3]. The decreased 11-Beta-hydroxylase (CYP11B1) activity in the zona fasciculata is responsible for the accumulation of 11-deoxycorticosterone (DOC), 11-deoxycortisol and an excessive production of adrenal androgens. Thus, the clinical manifestations of 11β-OHD is characterized by hypertension and hypokalemia due to mineralocorticoid (DOC) excess, in addition to the virilization of female neonates due to androgen excess [4]. Despite the fact that 11β-OHD is the second most common form of CAH, it lacks systematic assessment in adulthood [4]. Herein, we describe clinical cases of CAH due to CYP11B1 mutation in a Tunisian family, in addition to the long-term evolution of the disease in three members of this family.

## Patient and observation

**Figure 1:**
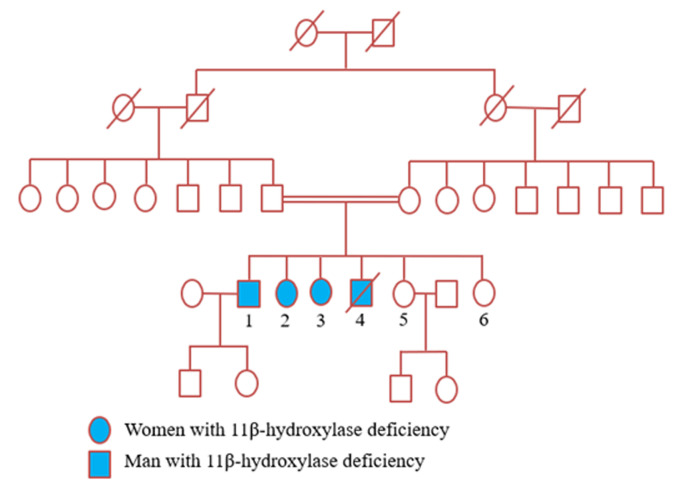
genealogic tree of a family with the 11β-hydroxylase deficiency

Four family members, originated from a commune in the Kairouan Governorate, Tunisia, were following in our departments for 11β-OHD (Figure 1).

**Patient 1:** a 37-year-old male (46, XY karyotype), issued from a consanguineous marriage, was diagnosed with 11β-OHD at the age of three. The disease was revealed by peripheral precocious puberty with an increased penile size, pubic hair development and bone age advancement. Laboratory findings at diagnosis was characterized by high serum concentrations of 11-deoxycortisol and adrenocorticotropic hormone (ACTH) with low plasma renin activity (Table 1). Genetic analysis found a homozygous p.Gly379Val (c.1136G>T) mutation in exon 7 of the CYP11B1 gene. During his follow-up, grade 3 hypertension was discovered at the age of 25 associated with hypokalemia treated by Calcium channel blocker (amlodipine 10 mg q.d.) and Beta-blocker (atenolol 50 mg q.d.). It has been complicated only by hypertensive retinopathy grade 1. The patient was treated by hydrocortisone 25mg b.i.d. Acute adrenal deficiency didn´t occur during the follow-up despite bad compliance of the treatment. He presented some episodes of abdominal pain that was alleviated after the correction of hypokalemia. Spironolactone 50 mg q.d. was prescribed later, at the age of 36, due to persistent hypokalemia. Progressive weight gain was observed since the age of 25 passing from 64 Kg to 90 Kg at the age of 36. He had a metabolic syndrome with an android fat distribution (waist circumference = 102 cm) and low HDL level (Table 2). External genitalia examination and bone density were normal. The patient was married and he had two healthy children aging respectively of three and one-year-old.

**Table 1:** patient´s 1 baseline biochemical parameters at diagnosis of the 11β-hydroxylase deficiency

Parameters	Patient 1	Reference range
Cortisol (ng/mL)	25	75-220
ACTH (pg/mL)	1500	10-50
11-deoxycortisol (nmol/L)	656	1.4-5
Plasma renin concentration (ng/L)	2.6	5.1-38.7
Serum aldosterone concentration (ng/L)	60	30-350
17-OH Progesterone (ng/mL)	135	0.5-2.4

**Table 2:** clinical and biochemical parameters of three patients with 11β-hydroxylase deficiency at the last visit

Parameters	Patient 1	Patient 2	Patient 3
Weight (Kg)	90	86	57
Height (cm)	169	154	153
BMI (Kg/m^2^)	31.5	36,26	24.35
Blood pressure (mm Hg)	140/70	130/80	170/110
Fasting plasma glucose (mmol/L)	4.7	4.3	4.26
Serum sodium (mmol/L)	140	141	136
Serum potassium (mmol/L)	3.1-3.7	3-3.5	2.87-3.4
Serum creatinine (μmol/L)	58	48	43
Total cholesterol (g/L)	1.17	1.12	1.33
HDL-cholesterol (g/L)	0.23	0.27	0.55
LDL-cholesterol (g/L)	0.84	0.77	0.7
Triglyceride (g/L)	0.62	0.44	0.39

**Patient 2:** a 35-year-old female (46, XX) was diagnosed with 11β-OHD at the neonatal age. The disease was revealed by the virilization of the external genitalia which was corrected surgically. The patient had a normal blood pressure during her regular check-up despite the presence of hypokalemia (Table 2). She was treated initially by hydrocortisone 30 mg b.i.d. and she didn´t have during her follow-up an acute adrenal deficiency. She had metabolic syndrome, class I obesity and polycystic ovary syndrome (PCOS) since the age of 28. The diagnosis of PCOS was considered regarding the presence of spaniomenorrhea, clinical (hirsutism, alopecia) and biological (testosterone level = 6.2 ng/ml) hyperandrogenism. The patient´s weight increased from 75 Kg at the age of 28 to 94 Kg at the age of 32, date of which the dose of hydrocortisone was decreased to 20 mg b.i.d. She had since lost 8 Kg and had normal menstrual cycles. Her bone density was normal two years ago. Spironolactone was also introduced due to persistent hypokalemia.

**Patient 3:** a 33-year-old female (46, XX) was diagnosed with 11β-OHD prenatally. Her mother was treated during pregnancy with dexamethasone. However, since she had also virilization of the external genitalia, she was operated on at the neonatal age. Genetic analysis found the same mutation as her brother, patient 1. The patient had an irregular follow-up and she was treated initially by hydrocortisone 30 mg b.i.d. She didn´t have a history of acute adrenal deficiency. Her menarche was at the age of 12. She had PCOS with hirsutism and irregular cycles, treated by estrogen therapy (estradiol 2 mg q.d.) associated with cyproterone acetate 50 mg q.d. At the age of 32, the patient was admitted for sore throat and repeated vomiting. Her body temperature was 38°C. She had a normal body mass index and a blood pressure of 120/90 mm Hg. She received amoxicillin 3 g t.i.d. for tonsillitis. Biochemical analysis revealed hypokalemia (2.7 mmol/L) without electrocardiographic changes. She was treated initially by intravenous potassium chloride then by an oral solution. Hypokalemia persisted after the withdrawal of potassium substitution and 24-hour ambulatory blood pressure monitoring revealed a mean systolic blood pressure of 169 mm Hg and a mean diastolic blood pressure of 116 mm Hg. The patient was discharged later with spironolactone 50 mg q.d.

**Patient 4:** a male patient diagnosed also with 11β-OHD. The disease was revealed at the age of 2 by peripheral precocious puberty. He didn´t have a genetic analysis. He died at the age of 12 by a postoperative infection following an orthopedic surgery for scoliosis. The remaining family members were not known to have CAH and they have not yet benefited from genetic analysis. His sister (patient 5), aged 28, married, mother of a boy and a girl, all in good health. The other sister (patient 6), 21 years old, was following in psychiatry for depression.

## Discussion

11β-OHD, like 21-OHD, has 2 forms. The classic form is characterized by peripheral precocious puberty and virilization in newborn females while the non-classic form, which is rarer, is revealed by hyperandrogenism during childhood [5]. Impairment of CYP11B1 function causes an elevated DOC levels leading to hypertension. High ACTH level and intact 17a-hydroxylase activity stimulate the androgen synthesis pathway and leads to androgen excess [4,5]. In 11β-OHD, despite the low cortisol level, the risk of adrenal crisis is known to be lower than in 21-OHD. However, in all patients with the classic form, increasing glucocorticoids doses are required in case of acute illness [1,4]. In the studied family, all patients had the classic phenotype of 11β-OHD. No adrenal crisis were documented during their follow-up. Hypertension was found in two from the four patients with 11β-OHD and virilization of external genitalia was found in the two females. The correlation between phenotype and genotype in 11β-OHD is not yet fully established [2,6]. More than 100 mutations in CYP11B1 gene were reported [1]. The most frequent mutation found in Tunisian patients was p.Gln356X [2]. In our case, a homozygous p.Gly379Val mutation in exon 7 of the CYP11B1 gene was determined.

The p.Gly379Val mutation has been reported also in a Tunisian study including 15 unrelated patients. The consanguinity rate in their families was high (12/15 families) and they are originated from five different regions of Tunisia. Only two classic 11β-OHD mutations were found: 11 patients with homozygous p.Gly379Val mutation in exon 7 and four patients with homozygous p.Gln356X mutation in exon 6 [7]. Another cohort of 108 genotyped patients with 11β-OHD from 11 countries showed that most cases were from North Africa and the Middle East. Among these patients, 32 were originated from Tunisia with 90.6% of them were Arab-Berber from Kairouan Governorate. 30/32 of the Tunisian patients had a homozygous p.Gly379Val mutation. Genetic finding showed also a homozygous Ser217Ilefs*42/ Ser217Ilefs*42 mutation and Gly379Val/ND mutation (ND: not determined) [6]. Other mutations were described also in the Tunisian population which are the c.652_653insT and the missense mutation Ser217Ile [2]. Patients with CAH were found to have a higher prevalence of metabolic and cardiovascular risk factors [8]. In 11β-OHD, cardiovascular risk was higher comparing to patients with 21-OHD, however, the mortality rate was lower since adrenal crisis were rarer [1].

The p.Gly379Val mutation was reported to be associated with mild hypertension and bone age advancement [6]. However, in the studied family, grade 3 hypertension was detected in two of the four cases with 11β-OHD. The treatment of CAH consists of glucocorticoids supplementation to reduce levels of excess androgens and mineralocorticoid precursors [3]. However, poorly controlled hypertension, as in both cases, may require the addition of an anti-hypertensive medication such as calcium channel blocker or potassium-sparing diuretic. End-organ damage secondary to severe hypertension such as hypertensive retinopathy, which was detected in patient 1, was also described in the literature [1]. Little is known regarding the prevalence of type 2 diabetes and bone mineral density in patients with 11β-OHD. Impaired final height was noted but good compliance to glucocorticoids could have a positive effect on final height [1]. In our family, glycemia and bone mineral density were in the normal range. Impaired final height was also found due to the bad compliance of the treatment.

Infertility is least likely to occur in patients with 11β-OHD or salt-wasting CAH. Conception without specific fertility intervention was reported in less than 10% in classic CAH and successful pregnancy was reported only twice in 11β-OHD [9]. In our cases, the two females with 11β-OHD were single. Prenatal treatment was superimposed on 21-OHD [4]. Cerame *et al*. (1999), reported the case of a newborn female with normal external genitalia after administering dexamethasone to the mother [10]. In the case of patient 3, although her mother received dexamethasone during her pregnancy, she had virilization of the external genitalia.

## Conclusion

In this paper, we reported the clinical and biological characteristics, treatment, and outcome of patients of the same family diagnosed with the classical form of 11β-OHD with p.Gly379Val mutation in the CYP11B1 gene. This disorder is insufficiently studied and frequently assimilated to 21-OHD. Additional investigations are required to better understand phenotype-genotype correlation, in addition to the evaluation of the long-term outcomes in a larger-scale studies.
